# Scaling-up the delivery of dog vaccination campaigns against rabies in Tanzania

**DOI:** 10.1371/journal.pntd.0010124

**Published:** 2022-02-10

**Authors:** Maganga Sambo, Elaine A. Ferguson, Bernadette Abela-Ridder, Joel Changalucha, Sarah Cleaveland, Kennedy Lushasi, Geofrey Joseph Mchau, Alphoncina Nanai, Hezron Nonga, Rachel Steenson, Paul CD Johnson, Katie Hampson

**Affiliations:** 1 Environmental Health and Ecological Sciences Department, Ifakara Health Institute, Ifakara, Tanzania; 2 Boyd Orr Centre for Population and Ecosystem Health, Institute of Biodiversity, Animal Health and Comparative Medicine, College of Medical, Veterinary and Life Sciences, University of Glasgow, Glasgow, United Kingdom; 3 Department of the Control of Neglected Tropical Diseases, Geneva, Switzerland; 4 Global Health and Biomedical Sciences, School of Life Sciences and Bioengineering, Nelson Mandela African Institution of Science and Technology, Arusha, Tanzania; 5 Ministry of Health, Community Development, Gender, Elderly and Children, Dodoma, Tanzania; 6 Department of Neglected Tropical Diseases, World Health Organization, Country Office of Tanzania, Dar es Salaam, Tanzania; 7 Directorate of Veterinary Services, Ministry of Livestock Development and Fisheries, Dodoma, Tanzania; Faculty of Science, Ain Shams University (ASU), EGYPT

## Abstract

An increasing number of countries are committing to meet the global target to eliminate human deaths from dog-mediated rabies by 2030. Mass dog vaccination is central to this strategy. To interrupt rabies transmission from dogs to humans, the World Health Organization recommends that vaccination campaigns should be carried out every year in all dog-owning communities vaccinating 70% of their susceptible dogs. Monitoring and evaluation of dog vaccination campaigns are needed to measure progress towards elimination. In this study, we measured the delivery performance of large-scale vaccination campaigns implemented in 25 districts in south-east Tanzania from 2010 until 2017. We used regression modelling to infer the factors associated with, and potentially influencing the successful delivery of vaccination campaigns. During 2010–2017, five rounds of vaccination campaigns were carried out, vaccinating in total 349,513 dogs in 2,066 administrative vaccination units (rural villages or urban wards). Progressively more dogs were vaccinated over the successive campaigns. The campaigns did not reach all vaccination units each year, with only 16–28% of districts achieving 100% campaign completeness (where all units were vaccinated). During 2013–2017 when vaccination coverage was monitored, approximately 20% of vaccination units achieved the recommended 70% coverage, with average coverage around 50%. Campaigns were also not completed at annual intervals, with the longest interval between campaigns being 27 months. Our analysis revealed that districts with higher budgets generally achieved higher completeness, with a twofold difference in district budget increasing the odds of a vaccination unit being reached by a campaign by slightly more than twofold (OR: 2.29; 95% CI: 1.69–3.09). However, higher budgets did not necessarily result in higher coverage within vaccination units that were reached. We recommend national programs regularly monitor and evaluate the performance of their vaccination campaigns, so as to identify factors hindering their effective delivery and to guide remedial action.

## Author summary

Globally there are approximately 59,000 annual human rabies deaths, with more than 99% of these resulting from dog-mediated rabies. Mass dog vaccination is known to be the cornerstone of effective control of dog rabies. Empirical and theoretical evidence shows that annual vaccination campaigns need to achieve 70% coverage of the susceptible dog population to interrupt transmission and ensure the dog population is protected until the next campaign. Recently, international organizations announced their commitment to reaching the global target of zero human deaths from dog-mediated rabies by 2030. Scaling-up of vaccinations is now underway in more than 100 rabies-endemic countries. However, there are operational and logistical challenges associated with scaling-up these operations in different geographical and cultural settings. This study monitored and evaluated the performance large-scale vaccinations in Tanzania. We found that vaccinations were not implemented in all villages, which resulted in coverage gaps. Additionally, vaccinations were not implemented annually and, as a result, coverage dropped below the critical vaccination threshold. We conclude that there is a need to improve the delivery of dog vaccinations for achieving the global target of zero dog-mediated human rabies deaths by 2030.

## Introduction

Each year, canine rabies causes approximately 59,000 human deaths worldwide, mostly in Asia and Africa [1]. Ninety-nine percent of these deaths result from rabies spread by domestic dogs [2]. Human rabies deaths are 100% vaccine-preventable through two complementary interventions: administration of post-exposure prophylaxis (PEP) to people bitten by suspected rabid animals to prevent disease onset; and sustained mass dog vaccinations (MDV) to eliminate transmission within the main source (reservoir) of infection [3]. It is estimated that more than 29 million patients are administered with PEP annually [1]. While human rabies can be effectively prevented with PEP, the intervention is expensive, with direct expenditure on PEP estimated at 1.70 billion US Dollars (US$) per year and indirect costs estimated at US$1.31 billion [1]. Both the disease and the financial burden of rabies falls disproportionately upon people living in rural communities where most rabies occurs. A case study in Tanzania estimated that a patient in a rural area, where most people live on less than US$1.25 per day, would need to spend over US$100 to access and complete the World Health Organisation (WHO) recommended PEP regimen [4]. Many families struggle to obtain PEP, either because they cannot afford it, or because PEP is out of stock locally. These barriers lead to poor compliance with PEP, delays in presentation to health facilities, and increased risk of death [5]. The recent inclusion of human rabies vaccine within the investment strategy of Gavi, the Vaccine Alliance, provides optimism that access to PEP for the poorest communities can be improved. However, human deaths will not be eliminated through strategies that rely on PEP alone, scaling up of MDV is also essential [6]. Investment in dog vaccinations can control rabies in the reservoir population and reduce exposures, thus protecting everyone in the community regardless of socio-economic status [7]. Economic analysis has shown that canine vaccination against rabies is also very cost-effective for preventing human rabies and is even cost-saving relative to PEP alone [8].

Dog rabies has been eliminated from industrialized countries in Europe and North America [9,10] and the continent-wide elimination of dog-mediated rabies from the Americas is now within reach [11]. Recent research has demonstrated that rabies can be eliminated through mass vaccination of dogs from even the poorest countries [12]. To draw attention to this neglected disease and efforts to control it, in 2020 the WHO and partners launched the “United Against Rabies forum”, to accelerate progress to reach “zero human rabies deaths by 2030”. To reach this target, more than 100 countries in which rabies is currently endemic need to implement nationwide MDV campaigns. Dog vaccinations across Latin America and the Caribbean, as well as pilot projects in different parts of the world such as Bangladesh, the Philippines, Sri Lanka, Vietnam and South Africa, have provided valuable lessons for successful delivery of MDV campaigns [13–16].

For the monitoring and evaluation of MDV performance several metrics can be considered, including the completeness of vaccination campaigns in terms of whether they reach all communities, the levels of vaccination coverage achieved within communities and the timeliness of campaigns. MDV campaigns should aim to reach all communities (campaign completeness), ensuring there are no areas left unvaccinated. Patchy coverage has been shown to facilitate persistence, since unvaccinated (incomplete) areas can be a source of ongoing transmission [17]. MDV campaigns should also aim to achieve 70% coverage of the susceptible dog population, a target that can interrupt the transmission cycle [18]. Finally, MDV campaigns should be conducted regularly within a target time interval (vaccination timeliness). Coverage declines rapidly following a campaign as vaccinated dogs die, susceptible (unvaccinated) dogs are recruited, and vaccine-induced immunity wanes (high-quality vaccines normally provide 2 to 3 year duration of immunity). If coverage of 70% is achieved during a campaign, then the next campaign should aim to be completed 12 months later to maintain sufficient herd immunity [19].

Rabies was first documented in Tanzania in 1932 [20]. Since then, the disease has spread widely throughout the country with varying patterns of infection between regions depending very much on the size of dog populations. As in most of the developing world, the domestic dog is the most important source of human rabies in Tanzania [21–23]. Rabies is endemic in the country and causes an estimated 552 (394–731) human deaths annually with at least 98% attributable to rabid domestic dogs [6]. The Tanzanian government through the Ministry of Livestock Development and Fisheries in collaboration with development partners had implemented dog vaccinations to control the disease [24–26]. However, these interventions have only covered a few districts and as a result, rabies remains uncontrolled across most of the country.

In 2010, Tanzania began the Rabies Elimination Demonstration Project, a large-scale intervention coordinated by the World Health Organization and funded by the Bill and Melinda Gates Foundation [24]. This project involved the first government-led large-scale dog vaccinations to be implemented across the southeast of the country. In this study, we evaluate the dog vaccinations delivery in southeast Tanzania through this project from 2010–2017, focusing on the coverage, completeness and timeliness achieved, and identifying factors associated with campaign performance.

## Methods

### Ethics statement

The study protocol was approved by the Medical Research Coordinating Committee of the National Institute for Medical Research of Tanzania (NIMR/HQ/R.8a/Vol.IX/2109), the Institutional Review Board of the Ifakara Health Institute and the Tanzania Commission for Science and Technology (COSTECH).

### Study sites

**Fig 1:**
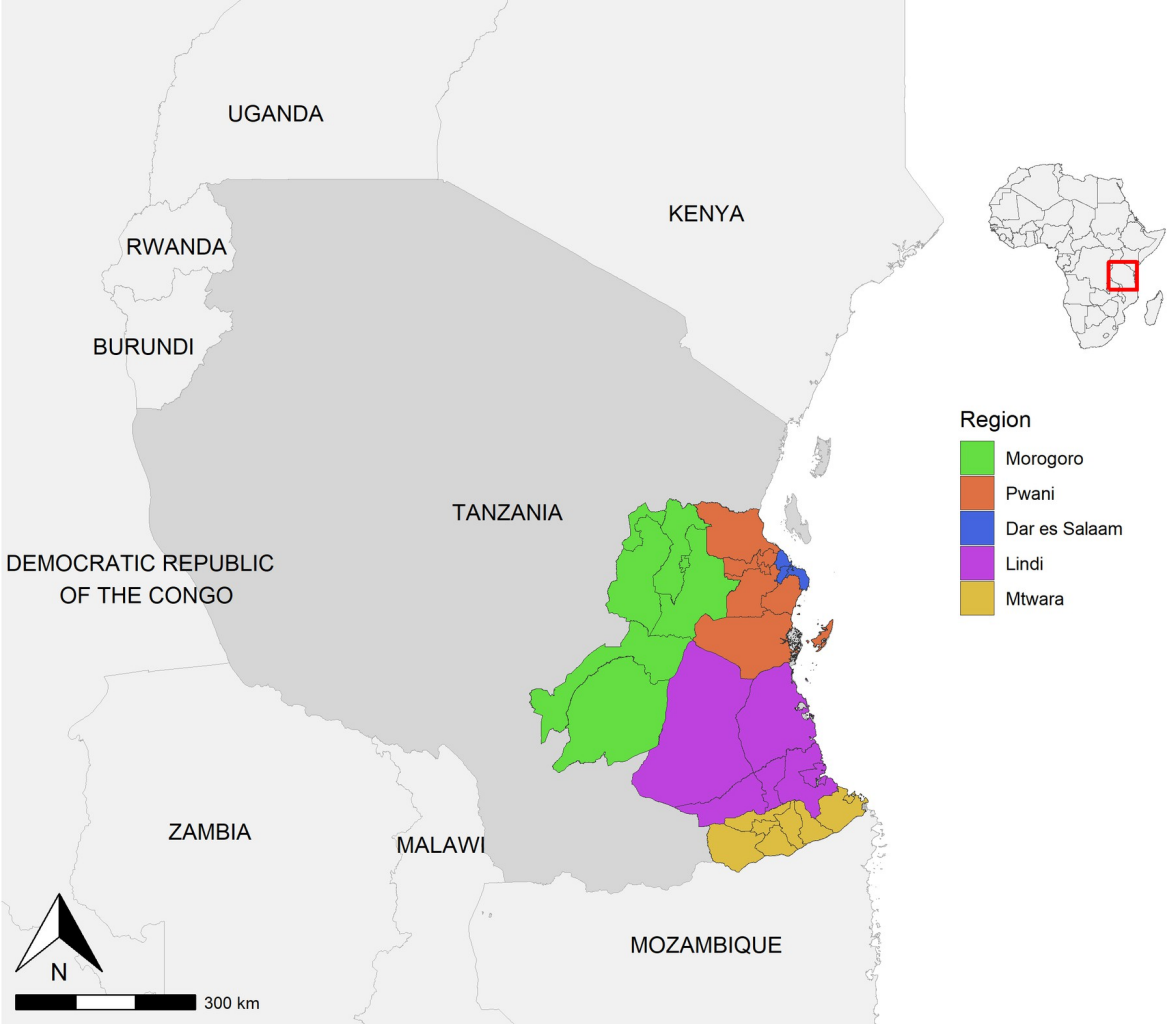
The study districts in south-east Tanzania where mass dog vaccinations and post-vaccination transects were undertaken. Shapefiles to make this figure were downloaded from the Diva GIS data portal (https://www.diva-gis.org/).

The study was conducted in 25 districts from five regions from south-eastern Tanzania: Lindi, Mtwara, Pwani, Dar es Salaam and Morogoro (Fig 1 and Table 1). The districts comprise rural, coastal and urban settlements, and cover an area of 160,000 km^2^, which represents 16% of the landmass of Tanzania [27]. According to the last official population census conducted in 2012, these districts had a population of about 8.5 million people, with an average annual population growth rate of 2.16% [27]. Over 60% of Tanzanians live in rural areas, depending solely on agriculture and fishing for their livelihood [27]. Those from urban areas are engaged in the civil service and small or large-scale business. These study districts were selected for this pilot project to exploit natural boundaries to facilitate the establishment and maintenance of a rabies-free area, including the coastline to the east, the Udzungwa Mountains to the northwest, and the Ruvuma River to the south. The Dar es Salaam–Mbeya highway to Morogoro and the railway line to Kilosa town define the northern boundary of the vaccination zone [24].

**Table 1:** Characteristics of the study districts where mass dog vaccinations and post-vaccination transects were conducted between 2010 and 2017.

Region	District	No of VUs	Mean distance (km) from HQ to VUs (SD)	Estimated dogs	Human population (1000s)	Human population growth (%)	Total number of households	Number of households owning livestock	_Area (km_^2^_)_	Budget for MDV (TZS 1,000,000s)	Number of vaccinators. (LFOs)
Dar Es Salaam	Ilala	26	10.5 (9.3)	8179	1,221	7	297,750	26,671	364.7	12.7	58
Dar Es Salaam	Kinondoni	34	9.6 (8.7)	8618	1,775	5	441,240	35,829	537.1	12.3	71
Dar Es Salaam	Temeke	30	8.1 (8.8)	5819	1,369	6	344,391	22,150	728.2	12.5	38
Lindi	Kilwa	100	56.5 (26.5)	4961	191	1	42,596	20,507	14545.4	8	29
Lindi	Lindi Rural	133	43.9 (14.7)	2788	194	-1	52,821	21,931	5971.5	8.6	69
Lindi	Lindi Urban	18	7.08 (7.8)	1876	79	7	22,344	6,500	1063	6.8	19
Lindi	Liwale	76	30.9 (26.9)	2386	91	2	21,084	10,582	15634	7.2	25
Lindi	Nachingwea	118	23.8 (23)	4477	178	1	48,145	21,726	5971.8	8.5	29
Lindi	Ruangwa	89	22.5 (12.2)	2095	131	1	37,326	17,605	2513.8	8.2	22
Morogoro	Kilombero	80	62.5 (49.8)	27599	408	2	93,331	34,427	7994.8	12.3	59
Morogoro	Morogoro Rural	140	41.7 (19.6)	12292	286	1	67,671	31,160	8267.7	11.8	100
Morogoro	Morogoro Urban	19	3.7 (3.2)	17476	316	3	76,039	12,060	288.3	11.8	50
Morogoro	Ulanga	65	44.8 (27.9)	19256	265	3	53,290	22,460	14476.8	12	41
Mtwara	Mtwara Rural	156	38.2 (18)	2635	228	1	58,602	21,651	3629.5	8.2	58
Mtwara	Mtwara Urban	15	5.7 (3)	1160	108	2	27,968	5,034	169.9	5.8	14
Mtwara	Nanyumbu	89	32.8 (13.5)	2364	151	1	40,746	13,474	5200.4	6.7	18
Mtwara	Newala	153	23.2 (12)	3630	205	1	58,035	28,279	1951.3	7.7	37
Mtwar	Tandahimba	155	20.4 (11.3)	1269	228	1	60,872	26,595	2047.3	7.3	33
Mtwara	Masasi	147	36.2 (14.6)	5027	248	1	67,872	30,565	4003.2	5.7	25
Mtwara	Masasi Urban	12	7.3 (5.6)	4504	103	2	28,222	28,279	752.8	5.7	21
Pwani	Kibaha Rural	50	35.9 (18.6)	9327	70	3	16,892	4,771	1500.8	10.2	25
Pwani	Kibaha Urban	53	13.7 (8.5)	6596	128	5	31,092	7,650	705.3	10.3	47
Pwani	Kisarawe	77	45.2 (23.1)	5010	102	1	25,475	12,275	4514.3	10,4	38
Pwani	Mkuranga	116	27 (13.3)	3891	223	2	51,101	17,610	2825.4	9.8	55
Pwani	Rufiji	115	45.9 (19.3)	3128	217	1	48,164	14,973	9383.8	10.5	29

SD = standard deviation; HQ = District headquarters; VU = Vaccination units; TZS = Tanzanian shillings; The exchange rate from Tanzanian shillings (TZS) to US dollars (US$) fluctuated from 1424 to 2300 between January 2010 and 2017 (https://www.xe.com/currencycharts/?from=USD&to=TZS&view=10Y); In this study, we use the average exchange rate of TZS 1876.29 to US$ 1.

### Data collection

#### Mass dog vaccination

Five rounds of MDV campaigns were carried out in the study districts between 2010 until January 2017. In each district, MDV campaigns were managed and supervised by the District Veterinary Officer or the Head of the Livestock Department, and Livestock Field Officers were assigned as vaccinators. In urban districts, MDV campaigns were carried out at ward level, while in rural districts they were carried out at the village level. In this study we refer vaccination unit as the area covered on the same day by a single vaccination team. Vaccination teams generally consist of a vaccinator, a recorder who document the details of vaccinated dog into the dog registers, and a village leader to assist with vaccination set up and dog owners on the vaccination process. The delivery of MDV campaigns was through a central vaccination point (CP) approach [28], whereby owners voluntarily brought their dogs to a centrally located vaccination point. These CPs were selected by livestock officers and village leaders. Each vaccination unit should ideally have one or more central vaccination points depending on their size, with vaccinators deciding whether they required more than one vaccination point per vaccination unit. In most cases, dog vaccinations began at 7 A.M. in the morning and lasted until 3.30 PM in the afternoon. Dog vaccines were provided at no cost to dog owners.

Prior to vaccinations, vaccinators communicated with local authorities to inform communities in advance of the campaigns (normally one week before with a reminder the day before the campaign). Communities were informed about the campaign (free dog vaccinations, place, time and the day of the campaign) using loud-speakers, fliers, announcements at schools and using community messengers urging dog owners to bring their dogs for vaccinations. On vaccination days, data on all vaccinated dogs were recorded in registers, including information about the dogs (name, age, sex), their owners (name and address (village)), the vaccination date, the location of the vaccination point and operating times. Dog owners were given vaccination certificates, and vaccinated dogs were fitted with plastic or fabric collars for the temporary identification of vaccinated dogs during post-vaccination transects.

#### Monitoring and evaluation

Transects have been conducted immediately after vaccination campaigns since 2013 [29]. Transects were walked (or occasionally cycled) on the same day as the campaigns from 4 to 6 p.m. when dogs were likely to be more active and visible, counting all marked (vaccinated) and unmarked (unvaccinated) dogs. In rural communities, transects were conducted in two randomly selected sub-villages in each vaccinated village (villages ranged in size from two to ten sub-villages, with a median of 4 sub-villages/village), aiming to representatively sample coverage within each village. In the first sub-village, enumerators were instructed to start transects at the center of the sub-village heading to the outskirts, while in the other sub-village, transects started from the edge of the sub-village and headed toward the center. Each transect was conducted by one enumerator for 1 h, therefore, taking a total of 2 hours to complete each village. In urban areas, enumerators were required to cover the jurisdiction of a street (a geographical area defined from the National Census, which covers a neighbourhood with several roads). Enumerators selected the direction at the start of transects, at the border of sub-villages/streets and at road junctions by spinning a pen. Prior to transects, training sessions were held with enumerators who were recruited from the community by village leaders. The recruited enumerators were respected and trustworthy members of the community who were familiar with village boundaries and have basic literacy and numeracy skills. Printed protocols and data collection forms were provided to enumerators during this training. Transect data were used for calculating vaccination coverage as described below.

#### Other contextual variables

We chose several variables that we hypothesized might influence campaign completeness and coverage, summarised in Table 2, and outlined briefly below.

**Table 2:** Showing summary of data from 2,066 vaccination units over a 7-year period showing explanatory variables hypothesised to be associated with completeness and coverage. Statistics reported separately for vaccination units with and without completed vaccination campaigns, and with low and high coverage respectively. All these statistics were calculated at the vaccination unit-level. N = number; TZS = Tanzanian shillings; VU = vaccination unit; HQ = headquarters.

	Completeness (N = 8959 units over 5 vaccination rounds)		Coverage (N = 6198 units with vaccination campaigns over 5 rounds)	
Predictors	No vaccination (N = 1548)	Vaccination (N = 7411)	Low coverage <70% (N = 3677)	High coverage ≥ 70% (N = 2521)
Cost (in TZS) per head per vaccination unit, Median (Range)	43.97(6.94–145.64)	43.98 (6.94–145.64)	41.28 (6.94–145.64)	44.68 (6.94–145.64)
Distance (km) between district HQ and vaccination unit’s centroid, Median (Range)	27.69 (0.20–171.92)	30.00 (0.20–171.92)	29.52 (0.20–171.92)	28.80 (0.20–152.81)
No. of estimated dogs per VU, Median (Range)	21.000(5.00–1121.00)	34.00 (5.00–3129.00)	28.00 (5.00–3031.00)	37.00 (5.00–3129.00)
Geographical area of vaccination units in km^2^, Median (Range)	14.123(0.08–10727.90)	23.22(0.08–10727.90)	19.36 (0.08–10727.90)	23.23 (0.08–7567.11)
Number of vaccination units, Median (Range)	116.00(12.00–156.00)	115.00 (12.00–156.00)	118.00 (12.00–156.00)	100.00 (12.00–156.00)
Number of people per vaccination unit, Median (Range)	1223.00(114.00–85735.00)	1566.00 (128.00–106946.00)	1517.00(114.00–106946.00)	1543.00(128.00–106946.00)
Type: Urban (vs Rural), N (%)	112 (7)	466 (6)	299 (8)	163 (6)
Prop. of households owning livestock, Mean (SD)	39.6 (11)	40.3 (10.6)	39.7 (11.3)	40.2 (10.2)
Season: Wet (vs Dry), N (%)	741 (48%)	4190 (57%)	2230 (61%)	1435 (57%)

We extracted demographic and geographical data from the 2012 national Population and Housing Census [27] to obtain the geographical area and human population size for each vaccination unit. We also extracted district-level population sizes, annual population growth rates, average household sizes (persons/household) and numbers of livestock keeping households. Human population numbers from 2010 to 2017 were projected backwards and forwards from the 2012 census using the district-specific annual growth rates (Table 1). The straight-line distance between the vaccination unit and district headquarters (where the district veterinary office or livestock office are located) were calculated from their centroids. We used the date of vaccination campaign to decide whether the campaign was conducted in the dry or wet season [30]. We assigned all vaccination campaigns between March and September including transition months (i.e. from the dry to the wet season, July to September) as dry season and the remaining months as wet season.

The budget allocated to dog vaccination in each district comprised direct expenses, categorised into: vaccines, consumables (syringes and needles), allowances for vaccinators (to cover food, subsistence and accommodation), advertisements, fuel, and administrative costs (for training, venue hire, training materials). The costs for dog vaccines and consumables were not included as they were procured internationally by WHO headquarters that arranged shipment to the WHO country office in Tanzania [24]. We note that WHO procured high quality the World Organisation for Animal Health (OIE) approved vaccines that are indicated to provide 3 years of immunity. We also excluded research costs associated with data gathering and evaluation. The other four cost items were known at the district level and validated at the WHO country office. In each district, we divided the total cost (excluding vaccines and consumables) by the total number of people in each vaccination unit to get the cost per human head per vaccination unit for each round of vaccination. The number of people in each of the vaccination units was obtained from the national census [27]. All dog vaccination costs were expressed in Tanzanian shillings (TZS) and converted to US dollars (US$) using the mean exchange rate between 2010–2017, which was 1 US$ to TZS 1876.29.

### Statistical analysis

The aim of this analysis was to determine if the vaccination campaigns achieved their intended goals, i.e. for annual vaccination campaigns in every vaccination unit to each achieve dog vaccination coverage of at least 70%, and to learn about factors associated with a binary outcome (success/failure) in achieving these vaccination goals.

*Vaccination completeness* was measured as the proportion of vaccination units in which campaigns were conducted in each district during each round of vaccination. During the initial campaigns (i.e. round one and two), data collection protocols were not well understood by district officials, and numbers of vaccinated dogs were aggregated at district level instead of at the vaccination unit for nine districts (round 1); and six districts (round 2). We therefore dropped these districts from our completeness analysis for these rounds.

*Vaccination coverage* was either estimated directly where transect data were available for a vaccination unit or estimated indirectly where transect data were not available. Direct estimates of coverage were estimated as vaccinated (collared) dogs divided by total dogs (observed either with or without collars). Indirect estimates were based on dogs vaccinated divided by the dog population estimated for that vaccination unit. Provided that the number of vaccinated dogs observed during a transect was non-zero, and that >5 total dogs were observed, the dog population at the time of the transect was estimated as per Sambo et al. [31]:

$$D=\frac{V_{d}}{\left(\frac{M_{d}}{\left(M_{d}+U_{d}\right)}\right)}*(1+PAR)$$

where D is dog population size, *V*_d_ is the number of vaccinated dogs from the vaccination records, *M*_*d*_ is marked dogs (with vaccination collars), *U*_*d*_ is unmarked dogs (unvaccinated and without collars), and PAR is the ratio of pups (<3 months) to adult dogs. The value of PAR that was used in this analysis was 0.256, estimated from a census of the dog population conducted in Serengeti district, Northern Tanzania between 2008–2016 [29]. Multiplication by (1+PAR) corrects the estimated dog population based on the assumption that vaccination campaigns fail to reach the majority of pups, and that without this correction, dog populations estimated in this way would be underestimated [31].

Transect-based dog population estimates were available for at least one vaccination round for >60% vaccination units, and up to three rounds of estimates were available in some cases. For indirect estimation of the dog population (where transect data were not available), we used three approaches: (1) If the missing population estimate lay between two transect-based estimates, an exponential dog population growth rate was estimated from these two estimates and used to project the population at the time of the intermediate vaccination campaign. (2) If there was only either a preceding or subsequent transect-based estimate, a human:dog ratio was calculated based on this estimate and the associated human population size projected from the 2012 human census and used to calculate the dog population at the time of the vaccination campaign assuming the same human:dog ratio. (3) If there were no transect-based dog population estimates for a vaccination unit, indirect estimates were obtained by taking the projected human populations at the times of the vaccination campaigns and dividing by district-level human:dog ratios [31]. In some cases, indirectly estimated dog populations led to vaccination coverage estimates that were greater than the proportion of adult dogs. Where this occurred, the population estimates were increased as necessary to keep coverage estimates below this maximum.

In addition to estimating campaign coverage, we estimated monthly vaccination coverage (in terms of the proportion of dogs with vaccine-induced immunity) from January 2010 to January 2017 in each district as follows:

$$V_{m}=max\left(N_{m},V_{m-1}e^{-(\frac{1}{v}+d)(\frac{1}{12})}min(1,\frac{D_{m}}{D_{m-1}})\right)$$

where *V*_*m*_ represents the number of vaccinated dogs at month *m*, *N*_*m*_ represents the number of newly vaccinated dogs in month *m*, and *D*_*m*_ is the dog population at *m* using the methods described above, *v* is the mean duration of vaccine-induced immunity (assumed to be 3 years [32]), and *d* = 0.595 is the annual dog death rate. This approach conservatively assumes both that dogs that were vaccinated previously are preferentially vaccinated in subsequent campaigns and that, if the dog population declines between months, then this is a consequence of an above average death rate, rather than a below average birth rate. Vaccination coverage through time in a district could then be estimated by summing *V*_*m*_ over all vaccination units in the district and dividing by *D*_*m*_ summed over the same vaccination units.

*Vaccination timeliness* was measured as the interval between vaccination campaigns. To maintain coverage above the critical immunity threshold (P_crit_) which is estimated at 20% [19], a 70% coverage at the time of the campaign (P_target_) is required. For MDV campaigns where 70% is not reached, coverage is likely to fall below P_crit_ with the risk that rabies transmission can be sustained. If 70% coverage is not reached, coverage above P_crit_ could also be maintained through implementing MDV with campaigns intervals of less than one year. We calculated the interval between campaigns (observed timeliness) and an ideal time interval (required timeliness) between campaigns based on the coverages achieved. Under the assumptions of a constant dog population and no existing immunity from preceding campaigns, a simplified equation to calculate vaccination coverage through time following a vaccination campaign can be derived [31].

$$P_{t}=P_{0}e^{-(1/v+d)t}$$

where *P*_*t*_ represents vaccination coverage at time *t* years after the preceding vaccination campaign, and *P*_*0*_ represents the vaccination coverage at the time of the preceding vaccination campaign. Using the above equation, we derived *t*_*crit*_, the campaign interval to prevent coverage falling below P_crit_:

$$t_{crit}=\frac{{log}_{e}\;(\frac{P_{0}}{P_{crit}})}{\frac{1}{v}+d}$$

We investigated eleven factors hypothesized to influence dog vaccination completeness and coverage (Table 2) by fitting them as predictors in binomial generalized linear mixed models (GLMM). We estimated the strength of their associations with completeness and coverage as odds ratios (ORs) with 95% confidence intervals. We did not examine timeliness in relation to these factors because timeliness generally related to vaccine availability, which was determined at the national and district level and not at the vaccination unit. The response variable for completeness was the binary outcome of whether a dog vaccination campaign was conducted (coded as 1) or not (coded as 0) in each of the vaccination. For coverage, the response variable was the joint outcome of vaccinated and unvaccinated dogs observed during the transect surveys (round 3 to round 5 of vaccination). Because many of the continuous predictors were right skewed, and because we generally expect multiplicative relationships between these predictors and completeness and coverage, we log-transformed all the continuous predictors by log_2_. Log-transformation to the base 2 was chosen to aid the interpretability of the odds ratios: an odds ratio is interpreted as the increase in odds associated with a doubling of the predictor. We carried out univariable regression analyses assessing the association with the response of each predictor in isolation, before fitting the predictors together in a multivariable model where their associations with the response would be mutually adjusted.

Before fitting models, we conducted data exploration to detect collinearity among the predictor variables using pairwise scatter plots and the variance inflation factor (VIF), as described by Zuur et al. [33]. Predictors with VIF values greater than or equal to 5 were assumed to be collinear and removed. This process was repeated until all VIF values were below 5. The resulting variables were fitted in a multivariable model, which was refined by a single round of model selection by dropping non-significant (P > 0.05) predictors. This single-step selection method was used to limit the biases and false positives associated with standard multiple stepwise model selection [34].

All models, including the univariable models, were adjusted for spatial (vaccination unit, district, and region) and temporal (vaccination round) effects. These factors were fitted as random effects, except for vaccination rounds, which was fitted as a fixed effect because it has too few levels (five in the completeness model, three in the coverage model) to be fitted as a random effect [35]. Additionally, an observation-level random effect to account for overdispersion was fitted in the coverage model [36]. The random effects and the fixed effect of vaccination rounds represented aspects of the study design rather than hypothesized correlates of completeness and coverage and were therefore not included among the variables subject to model selection.

All models were fitted using the *glmer* function of the *lme4* package [37]. All statistical analyses were conducted using *R* version 4.1.1 [38]. The data and code that support the findings of this study are openly available and can be obtained from Zenodo repository at https://doi.org/10.5281/zenodo.5854712.

## Results

**Fig 2:**
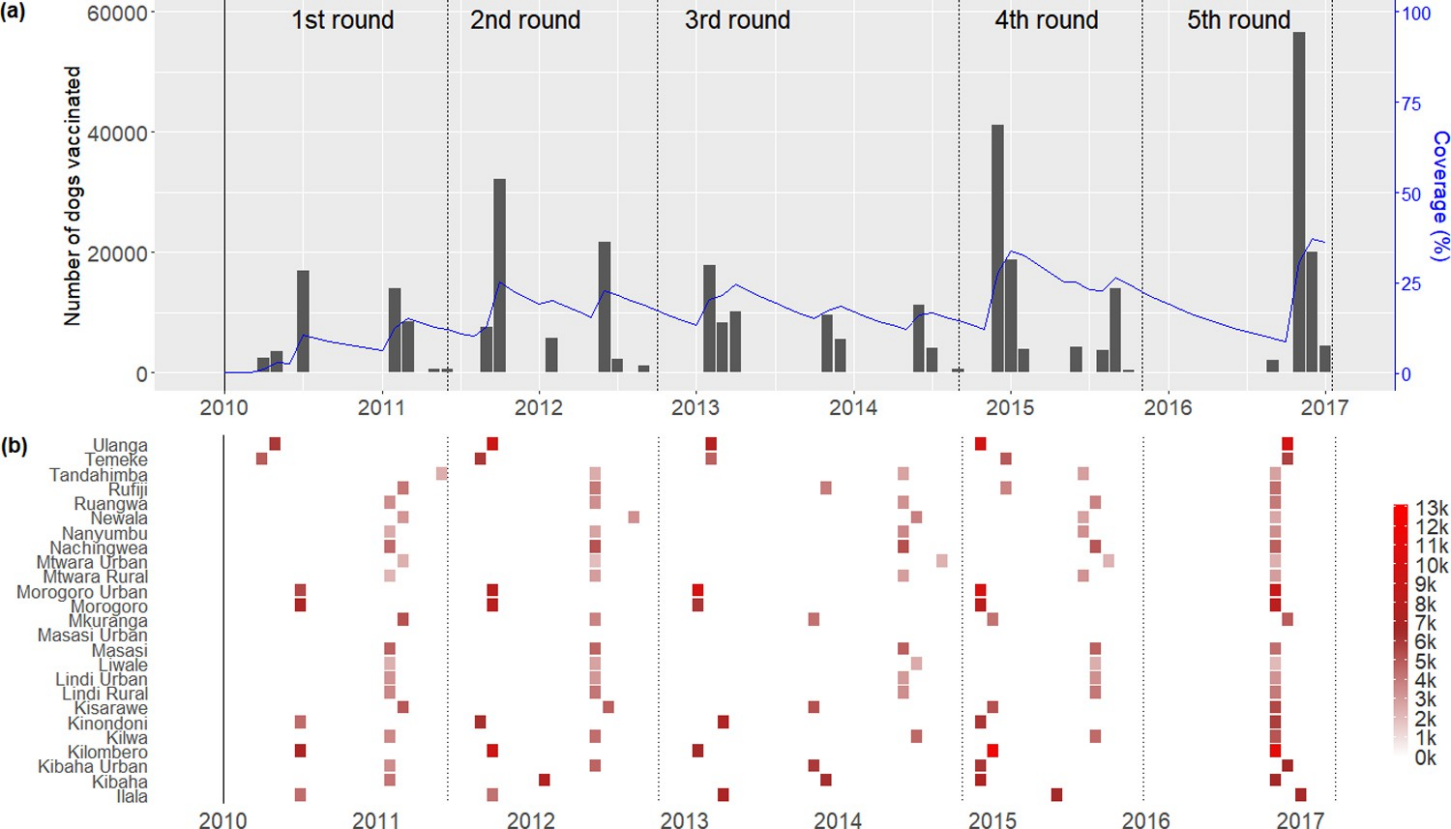
**(a) The number of dogs vaccinated and overall coverage across the study area from 2010 to 2017 and (b) detail on the number of dogs vaccinated in each district.** Coverage was calculated from the monthly estimated total number of vaccinated dogs divided by the estimated monthly total dog population estimate (blue line). The number of dogs vaccinated in each district each month ranged from 0 to 13 thousand dogs, indicated by the “heat intensity” of the colours from white to red (maximum).

From 2010–2017, campaigns were carried out that vaccinated 349,513 dogs in 2066 vaccination units resulting in data from a total of 8,959 vaccination units across the study period. The number of dogs vaccinated increased over the five rounds of campaigns but remained fairly stable from round four to five (Fig 2A). Initially, dog vaccinations were focused on urban areas and then expanded to rural areas. Vaccination campaigns greatly improved when transects were introduced in 2013 for monitoring vaccination programmes. Campaign monitoring helped in identifying areas where vaccination campaigns were poorly implemented or missed entirely.

Although campaigns were implemented in every district (Fig 2B), they were not completed in every vaccination unit (Table 3). However, completeness also generally improved from year to year. In the first round only 3 of 25 districts had vaccination campaigns in 100% of vaccination units, whereas 7 districts had campaigns completed in 100% of their vaccination units by the fifth round.

**Table 3:** Summary of the performance of vaccination campaigns in terms of completeness, coverage and timeliness for the 25 study districts over the five rounds of vaccinations from 2010–2017.

Performance indicator	Calculation	Round 1 (Mar 2010)	Round 2 (Jun 2011)	Round 3‡ (Sep 2012)	Round 4 (Aug 2014)	Round 5 (Sep 2016)
Number of vaccinated dogs	_	46,251	68,503	69,579	83,953	81,227
Completeness*	Prop. districts with 100% (90%) completeness Median (range) completeness	13% (20%) 77% (51–100%)	11% (26%) 78% (33–100%)	17% (42%) 84% (57–100%)	28% (64%) 95% (56–100%)	28% (68%) 96% (62–100%)
Coverage^⟢^	Prop. of districts with >70% coverage Median (range) coverage	N/A N/A	N/A N/A	60% 70% (58–78%)	26% 64% (46–80%)	30% 66% (31–84%)
Coverage	Prop. VUs with >70% (>60%) coverage Median (range) coverage	N/A N/A	N/A N/A	21% (37%) 53% (0–80%)	19% (35%) 55% (0–80%)	21% (38%) 56% (0–80%)
Timeliness	Prop. districts revaccinated in ≤13 (≤18) months Median (range) interval in months	N/A N/A	8% (100%) 16 (12–18)	0% (40%) 22 (16–27)	16% (72%) 15 (12–26)	4% (56%) 15 (13–24)

‡Based on five districts with transect data

*Excluding districts with incomplete data

^⟢^Coverage was calculated directly from the transect surveys and did not account for the pup:adult ratio (PAR); Coverage estimates exclude villages without transect data; N/A: either not applicable or no data and VU: vaccination units.

A total of 3777 post-vaccination transects were conducted in 1322 (64%) of the 2066 vaccination units between 2013 and 2017. Of these 3777 transects, 1439 (38.1%) counted fewer than five dogs. Of these 1439, 13.7% did not observe (count) any dogs (S1 Table).

**Fig 3:**
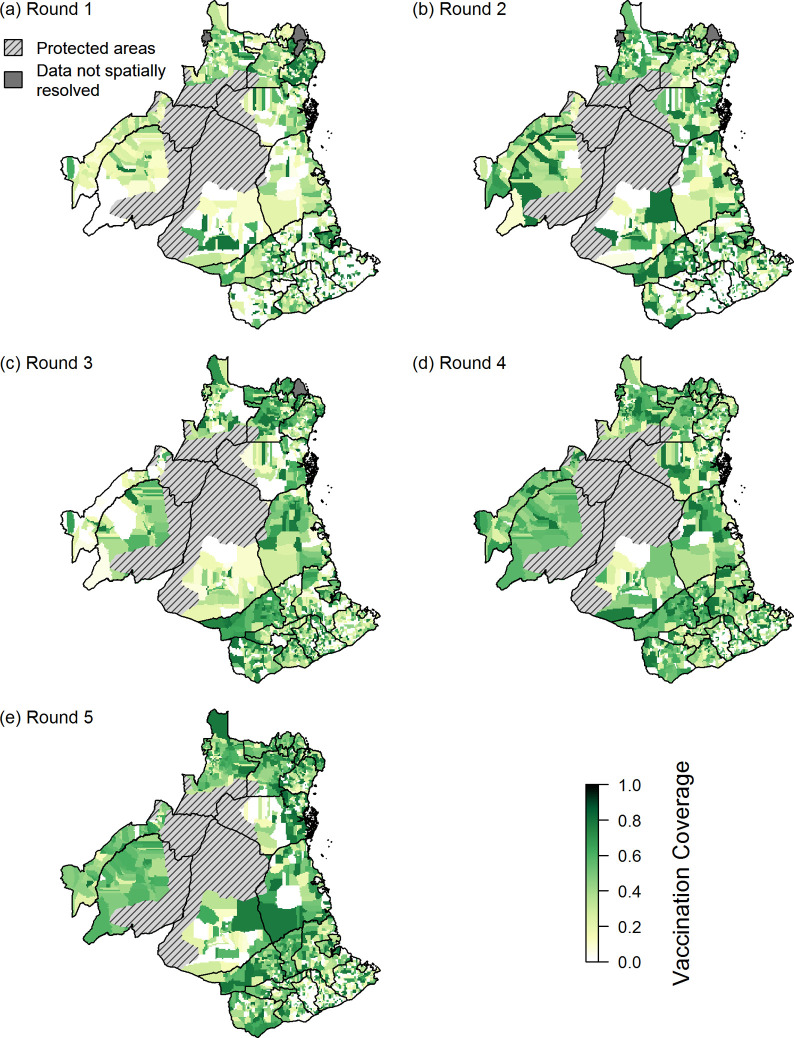
Coverage achieved in the five rounds of vaccination campaigns at the level of the vaccination unit. Coverage for rounds 1 and 2 was calculated using projected dog population estimates, while coverage for rounds 3–5 coverage were estimated as explained in the Methods. Darker green shading corresponds to higher coverage while white shading indicates that no vaccination campaign was undertaken. Hatched represents forest reserves or wildlife-protected areas. Shapefiles to make this figure were downloaded from the Diva GIS data portal (https://www.diva-gis.org/).

At the level of vaccination units, patterns of coverage varied markedly across the study area (Fig 3). Over the three rounds (3 to 5) when coverage was monitored, only 19–21% of vaccination units achieved the recommended coverage of 70% (Table 3).

**Fig 4:**
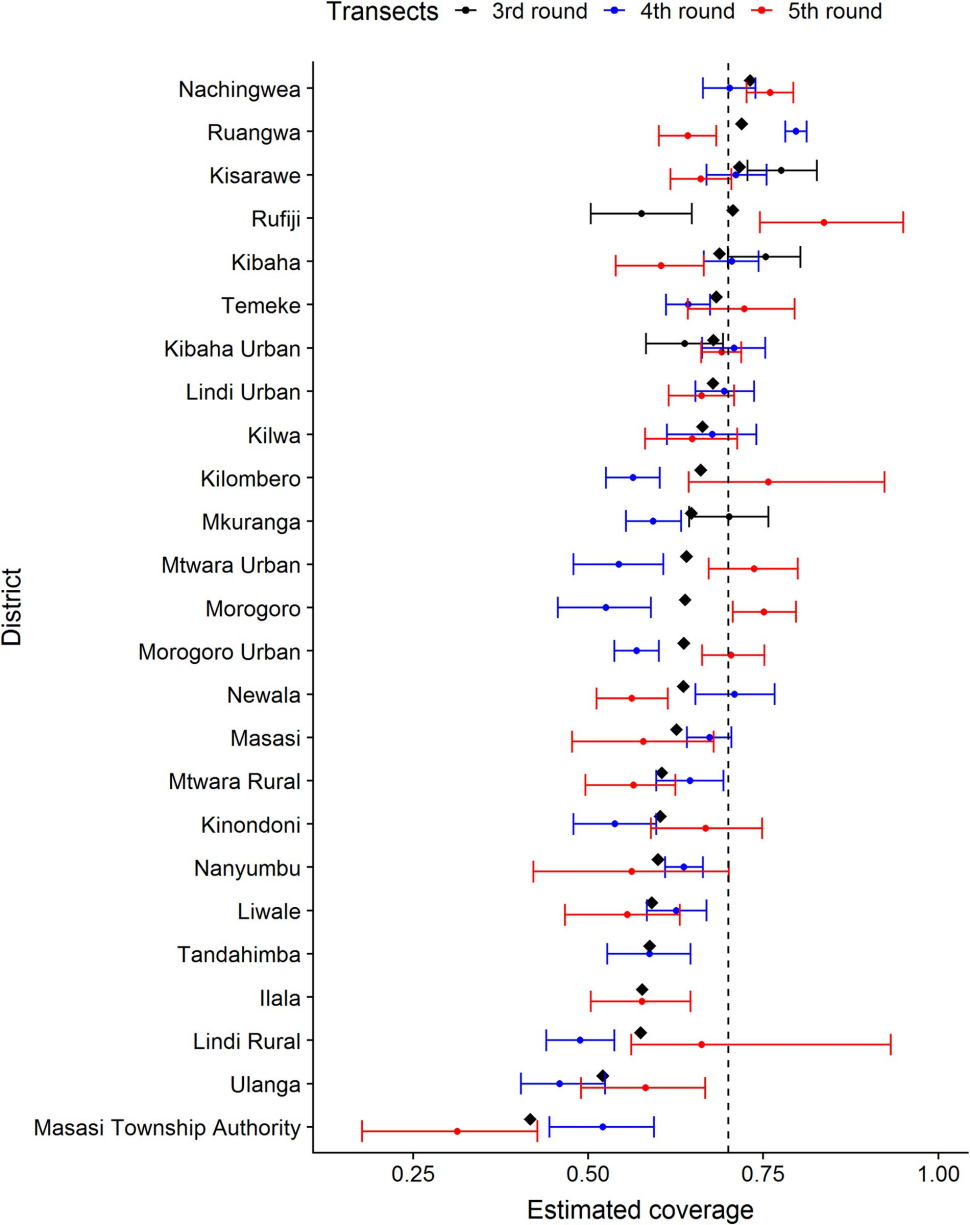
Coverage achieved during campaigns in each district from 2013 to 2017 (third to fifth round of vaccinations) calculated directly from transects without accounting for the pup:adult ratio (PAR) and excluding villages without transect data. Black diamonds represent mean coverage across the three rounds. The black dashed line represents the target 70% vaccination coverage threshold.

At the district level, overall, vaccination coverage increased with the implementation of campaigns (Table 3). Across the last three rounds district-level coverage ranged from 31% in Masasi Township to 83% in Rufiji (Fig 4), with only one of 25 districts consistently achieving average coverage of 70%.

**Fig 5:**
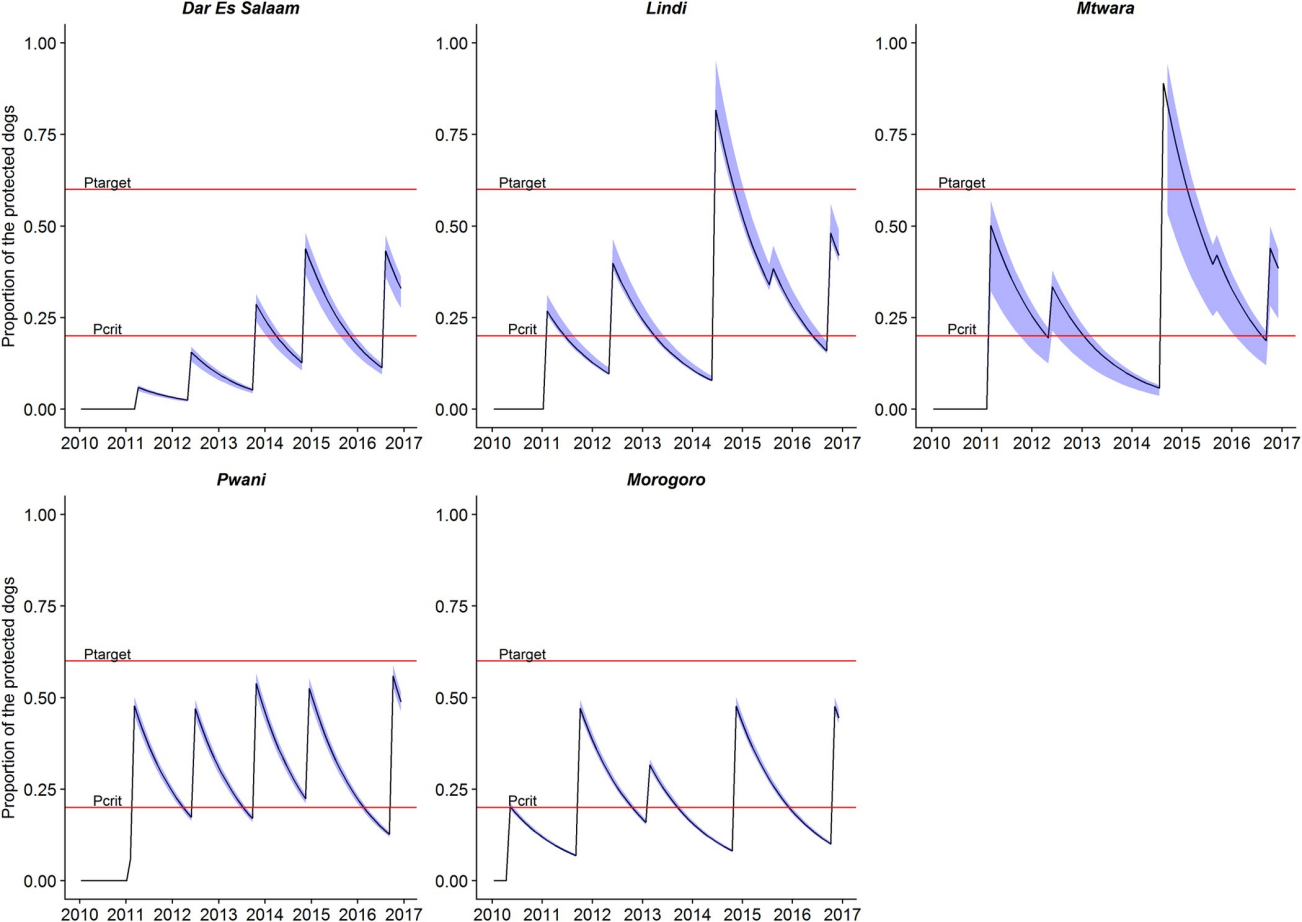
The campaign timeliness in each of the five regions from 2010 to 2017. The target coverage (P_target_) of 70% was not achieved in all regions (upper red line), and between campaigns coverage declined due to dog population turnover. When annual campaigns achieved high coverage, coverages were sustained above the critical immunity threshold (below the red line labelled P_crit_) for approximately 12 months. The time lag between campaigns (up to >20 months), caused coverage to decline below P_crit_. Mean coverage across all district in the regional was calculated from the number of vaccinated dogs in each region divided by the number of estimated dogs in each region.

When measured vaccination timeliness at district level, our result show that campaigns were not all completed at 12-month intervals, with considerable variation between rounds, resulting in lengthy declines in coverage between campaigns (Fig 5). For example, the median campaign interval between the first and second round of vaccination was 16 months and ranged from 12–18 months (Table 3). Delays to the second round (of up to 18 months) were caused by delays in releasing money to districts to enable them to conduct dog vaccinations as dog vaccines were available, remained from the initial purchase of vaccines [24]. When there were shortages of vaccines (i.e. the third round of MDVs), the reported median interval was even longer, at 22 months (range 16–27 months). MDV campaigns were conducted in a much more timely manner during the fourth and fifth round of vaccinations due to improved procurement.

**Fig 6:**
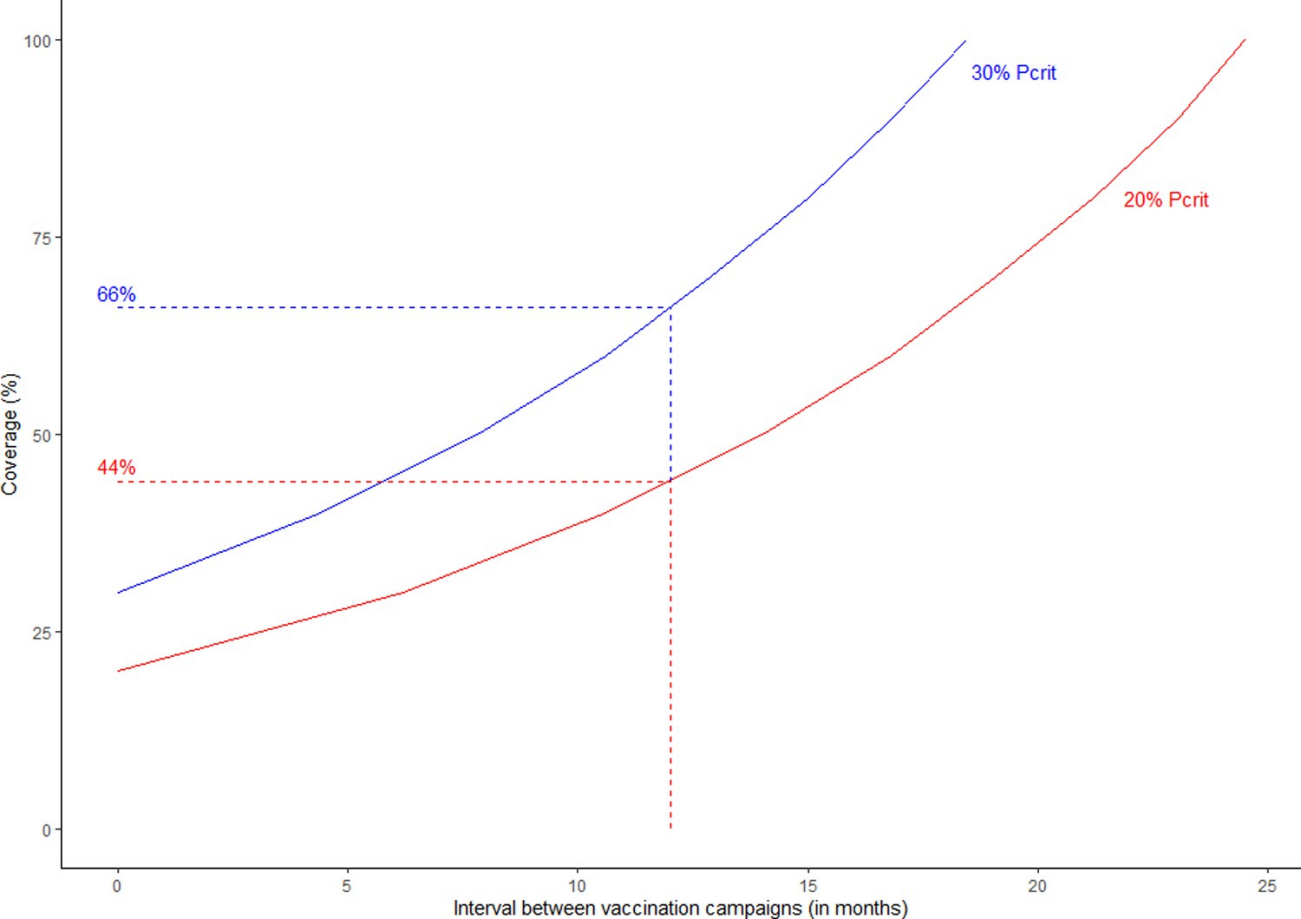
Required vaccination campaign intervals to prevent coverage falling to critical levels. For the populations in Tanzania, 66% coverage should maintain herd immunity above P_crit_ of 30% for 12 months, assuming the use of high-quality vaccines (as used in this study) where the average duration of vaccine-induced immunity is 3 years.

Our analysis showed that vaccination campaigns conducted annually would need to achieve at least 66% coverage to prevent coverage dropping below 30% before the next annual campaign (Fig 6). In a situation where less than 66% coverage was achieved, vaccination campaigns would need to be conducted more frequently. For example, if campaigns achieved 50% coverage they should be repeated within seven months. Ideal campaign intervals depend on the assumed value of P_crit_. For example, assuming a lower P_crit_ of 20%, campaign coverage of 44% should maintain herd immunity for 12 months.

In both the coverage and completeness models, the predictor variables ‘cost per head’ and ‘vaccinators per 1000 people’ were highly correlated, therefore we omitted vaccinators per 1000 people. After omitting vaccinators per 1000 people, the remaining predictors met the criteria for low multicollinearity, with VIFs in the range of 1–5. Four predictors that were not significant were dropped in the multivariable completeness model: number of villages, proportion of livestock owning households, setting (rural versus urban), and household size. For the coverage model, only four predictors were significantly associated with coverage and were retained in the model: the number of people in the vaccination unit, the estimated number of dogs per vaccination unit, human population size, and season.

In the multivariable regression analysis, we found that vaccination units with larger geographical area were associated with better campaign completeness (OR: 1.24; 95% CI: 1.19, 1.30; Table 4). Similarly, vaccination units further from the district livestock offices were associated with better campaign completeness (OR: 1.11; 95% CI: 1.04, 1.19; Table 4). Completeness in round 2 was significantly lower than in round 1, then increased significantly from round 2 to 3 and from 3 to 4, with no significant change from 4 to 5. Overall, completeness was substantially higher in rounds 4 and 5 than in rounds 1–3 (Table 4).

**Table 4:** Odds ratio (OR) estimates of characteristics of vaccination units (predictors) and the success of rabies vaccination campaigns in terms of coverage and completeness. OR and 95% confidence intervals (CIs) were estimated using binomial generalised linear mixed-effects models (GLMMs). Each predictor was fitted separately in univariable models, adjusted only for random effects and round, and combined in a mutually adjusted multivariable model. Non-significant predictors were removed from the model in a single round of model selection (see main text for details).

	Completeness OR (95% CI)		Coverage OR (95% CI)	
Predictor	Univariable	Multivariable	Univariable	Multivariable
Area (km^2^)‡	1.32 (1.27, 1.38)	1.24 (1.19, 1.30)	1.01 (0.99, 1.03)	-
Distance from HQ to the vaccination units (km)‡	1.26 (1.18, 1.34)	1.11 (1.04, 1.19)	1.05 (1.02, 1.08)	1.05 (1.02, 1.08)
Estimated dogs per vaccination unit‡	1.37 (1.29, 1.47)	1.12 (1.04, 1.20)	0.87 (0.85, 0.90)	0.86 (0.83, 0.89)
Cost per human head (Tanzanian shillings)‡	1.60 (1.19, 2.14)	2.29 (1.69, 3.09)	1.12 (0.98, 1.29)	-
Number of vaccination units per district‡	0.36 (0.21, 0.61)	-	0.94 (0.71, 1.24)	-
Household size‡	4.02 (0.33, 48.63)	-	1.13 (0.37, 3.47)	-
Number of people in vaccination unit‡	1.53 (1.40, 1.66)	1.41 (1.29, 1.54)	0.96 (0.92, 0.99)	1.04 (1.00, 1.09)
Proportion of households owning livestock‡	1.49 (1.04, 2.13)	-	1.02 (0.87, 1.21)	0.94 (0.79,1.12)
Category (urban versus rural)	0.64 (0.30, 1.37)	-	0.66 (0.49, 0.89)	-
Season (wet versus dry)	1.44 (1.17, 1.78)	1.40 (1.14, 1.72)	1.15 (1.01, 1.32)	1.17 (1.03, 1.33)
Second round	0.38 (0.31, 0.46)	0.43 (0.35, 0.54)	-	-
Third round	1.69 (1.37, 2.08)	1.78 (1.44, 2.20)	-	-
Fourth round	3.10 (2.48, 3.89)	3.50 (2.76, 4.43)	1.19 (1.09, 1.30)	1.20 (1.09, 1.31)
Fifth round	3.41 (2.71, 4.28)	2.94 (2.29, 3.77)	1.20 (1.09, 1.33)	1.10 (0.97, 1.25)

HQ: district headquarters

‡ these variables were log_2_-transformed, so odds ratios can be interpreted as associated with a twofold difference in the untransformed variable.

Our model investigating vaccination coverage indicated that there were significant differences in coverage between villages located near and far from district headquarters, with more distant villages having better coverage than those located near to headquarters (Table 4). However, the strength of the association was weak, with a doubling in distance being associated with only a 5% increase in the odds of a dog being vaccinated (OR: 1.05; 95% CI: 1.02, 1.08). The model indicated that vaccination units with more dogs had lower coverage than vaccination units with fewer dogs (OR: 0.86; 95% CI: 0.83, 0.89). The number of people (human population size) was positively, but weakly, associated with coverage (OR: 1.04; 95% CI: 1.00, 1.09). Additionally, campaigns conducted during the wet season achieved better coverage in vaccination units than those conducted during the dry season (OR: 1.17; 95% CI: 1.03, 1.33).

## Discussion

We evaluate the delivery of MDV campaigns across 25 districts in Southeast Tanzania using three operational indicators: completeness, coverage and timeliness, finding that most MDV campaigns did not meet recommended targets. For example, over five rounds of MDVs, campaigns were conducted in every vaccination unit in only 13–28% of districts, and at the district level, MDV campaigns achieved a mean coverage of 50%, below the WHO-recommended coverage of 70%. The intervals between campaigns were also not ideal, typically much longer than the recommended 12 months, likely reducing their effectiveness. For example, the median interval between the second and third rounds of vaccination campaigns was 22 months and ranged from 16–27 months. The relatively poor MDV performance demonstrates the considerable challenge of delivering MDV campaigns effectively at scale in areas without previous experience of undertaking MDV. However, almost all performance indicators improved considerably over time, which we attribute to both training and experience gained. Monitoring of MDV campaigns is also necessary to assess their performance and ultimately evaluate their effectiveness in controlling rabies. We found monitoring to be useful for detecting unvaccinated pockets missed by MDVs (low completeness) as well as communities with low vaccination coverage [29], which should be targeted for remedial vaccination. We recommend rapid assessment of dogs to ensure areas are not missed and campaigns achieve high coverage. Between 2010 and 2013 (when there were no campaign monitoring), dog vaccinations were concentrated in urban areas whereas hard-to-reach villages (remote areas) were generally missed out or achieved low vaccination coverage until when transect surveys were introduced in 2013.

In general scaling-up infectious disease control campaigns require coordination, infrastructure, and capacity for both timely procurements (or production) of vaccines/ drugs and effective delivery to communities in need. However, bureaucratic procurement and weak distribution systems were the greatest challenge to timely implementation [24], and are out of the control of practitioners, needing resolution at the programmatic level. In the discussion below, we expand on the operational lessons learnt from these MDV campaigns in Southeast Tanzania.

Our study investigated demographic, resource (human and financial) and geographical factors that affect MDV campaign completeness and coverage. We cannot modify geographical factors, but we can learn from geographical factors to improve the performance of the campaigns, for example, on where to increase vaccination points, and how to better mobilise people to improve participation. Our finding that vaccination units with more dogs had lower coverage could be linked to livestock keepers who tend to own more dogs than farmers [31]. In this regard, villages that were dominated by large number of pastoralists were prioritised by vaccinators because of their tendency to keep dogs, which might explain their positive relationship between completeness and vaccination unit area. Vaccination units from rural areas tend to be larger in size and contain more dogs than those from urban areas. However, the lower coverage in pastoralist communities (that largely depend on livestock for their livelihoods) may be because they are less able to bring their dogs to vaccination (central) points because they have to walk further and are less used to restraining their dogs [28]. Although we found that distance from district headquarters was associated with better coverage, the effect size was not large. Recent research from Malawi also reported that distance to the closest city had a negligible effect on coverage [39]. We also found that campaigns conducted during the dry season had lower completeness and coverage than those in the wet season. These findings were contrary to our expectation, as we assumed that during the dry season roads would be more passable and farmers would not be occupied by farming activities. The reason for this might have been that farmers were more involved in preparatory activities and pastoralists in transhumance movements which might have decreased coverage. However, the effect size of season on both completeness and coverage was small. Nonetheless, planning could ensure that the timing of campaigns considers agricultural cycles and transhumance movements.

Our study had several limitations. In most cases, we estimated vaccination coverage using transect data. Only observable dogs were counted from transects, which results in systematic bias [29,40]. Transects only allow dogs that are seen to be counted, but many dogs are hidden from view and a large proportion of our transects counted less than five dogs (S1 Table). Pups were less likely to be observed during transect surveys but we tried to adjust for this using the pup/adult ratio [31]. We were unable to investigate socioeconomic status or cultural factors, which can be barriers to dog vaccination [28,41,42]. We also did not investigate the role of religion. Fourteen (50%) of our study districts were island or coastal areas with predominantly Muslim communities (S1 Table), where dog ownership is less common [31,41,43]. This was the reason why our transects were frequently counting less than five dogs as these areas are dominated by coastal or Muslim communities (S1 Table). We did not evaluate the impact of these campaigns on controlling rabies, but rather focused on operational indicators. Despite all these challenges, analysis of the epidemiological impacts of these MDV campaigns showed that the domestic dog vaccinations were generally effective in reducing exposure risks in humans and decreasing rabies incidence [44].

We propose that feedback on the indicators for campaign completeness, coverage and timeliness be taken up directly by programme managers to address weak performance. If the target coverage is not reached, there are options to prevent coverage from falling below the critical immunization threshold: conducting biannual MDVs campaigns (i.e. at six months interval) or more immediate remedial vaccinations. Biannual campaigns would be both financially and logistically challenging in low-income countries like Tanzania. Remedial vaccinations to increase coverage in poor-performing areas or areas that were missed entirely is a rapid and targeted way to increase herd immunity and will give time for rabies project managers to prepare (i.e. procure and distribute vaccines) for the next annual vaccination campaign. Although feedback was provided to district veterinary officers to improve subsequent MDV rounds, this was not done in real-time to guide remedial vaccinations, which could have been done had the transect data been immediately entered via a mobile phone app as exemplified by other recent studies [26,45,46].

The greatest challenge to the implementation of MDV appears to be sustainable government-led funding. In most of the low-middle-income countries (LMIC), there is absence of a formal and budgeted programme with allocated funds for surveillance and dog vaccination interventions. Lower campaign budgets were associated with and potentially caused lower campaign completeness. A previous study also showed that remote locations were not reached when allocated budgets were smaller [41]. This was relevant to our findings; in Fig 3 lower levels of coverage were observed along the edge of the protected areas (Selous Game Reserve). These remote areas require more effort and planning to be effectively vaccinated.

The budgets we report on were used for logistic issues such as allowances to vaccinators and distributing vaccines to the communities. We found that districts that were less-resourced (with lower budgets) had lower completeness. However, budgetary constraints appeared to only affect completeness and not coverage. We found that this association was not only present in the univariable model but was strengthened by adjustment for other potentially confounding factors in the multivariable model. For scaling-up rabies elimination programmes, financial support for campaign logistics is needed.

The timeliness of MDV campaigns was also tied to the ability of the Ministry of Livestock to plan, procure and distribute dog vaccines and release funds to district officials. The original plan was to demonstrate the feasibility, sustainability, and cost-effectiveness of human rabies elimination through dog vaccination, thereby catalysing similar initiatives in Africa and Asia. The Tanzanian government was expecting to take over the project but failed to do so. Although the experience gained has provided capacity and there are a few districts where some limited vaccination has subsequently been conducted. The challenge is that no or insufficient vaccines have been procured by the central government and funds are not provided to support logistics of this work. In many LMICs, rabies is repeatedly ranked top priority zoonotic disease to be targeted for elimination, however ownership of MDV interventions is missing as there is inevitably inadequate funding allocated by LMICs for the control of rabies [47–49]. Countries starting to scale-up MDV campaigns must develop strong procurement systems for the management and timely distribution of vaccine for example OIE rabies vaccine banks that can bulk procure high-quality vaccines. Moreover, for the elimination of rabies, demonstrated funding commitments will be crucial, to allow countries to secure dog vaccines at affordable prices and sustain regular MDV campaigns to interrupt transmission [17,32].

## Conclusions and recommendations

As we are fast approaching the 2030 deadline for achieving “zero dog-mediated human rabies deaths”, it is essential for rabies-endemic countries to learn and promote the most efficient strategies to control and eliminate rabies. Our evaluation showed that this pilot in southeast Tanzania did not reach 100% of the project targets: campaigns were not conducted in all vaccination units, districts did not consistently achieve 70% coverage, and there were serious delays between vaccination campaigns. To increase completeness, managers must ensure that they monitor where campaigns are conducted and ensure vaccination units are not missed. To improve coverage, rapid assessment of dogs vaccinated in each unit can be used to inform remedial vaccination. Dog demographic data and coverage data can also help to forecast dog vaccine needs for future vaccination activities. We also propose that monitoring be conducted in real-time for example through post-vaccination transects, to identify areas with poor coverage or no MDV. Although this will require some resource, the rapid and targeted remedial vaccination is likely to be more efficacious and cost-effective in controlling rabies. We conclude that completeness, coverage and campaign timeliness are useful measures for monitoring and supporting progress towards rabies control goals.

## Supporting information

S1 Table The characteristics of the study districts on dog ownership. (DOCX)
