# Supplementary material for: Scaling-up the delivery of dog vaccination campaigns against rabies in Tanzania
Source: PLoS Negl Trop Dis. 2022 Feb 10;16(2):e0010124. doi: 10.1371/journal.pntd.0010124 (PMC8865671; doi:10.1371/journal.pntd.0010124)
Supplement: S1 Table — (DOCX) [file pntd.0010124.s001.docx]

**S1 Table. Showing the characteristics of the study districts**. Dog keeping is much less common in Muslim communities; in these areas, transect surveys were normally counting fewer dogs (<5) dogs per transect in a village. There is high variation of human to dog ratio between districts.

| Region | District | Total transects (Frequency of observing <5 dogs during transect surveys) | Rounds of transects | Setting | Dominated  religion | Human population  As per 2014 [1] | Dog population  [2] |
| --- | --- | --- | --- | --- | --- | --- | --- |
| Mtwara | Tandahimba | 266 (188) | 3 | Rural inland | Moslems | 232,087 | 6,365 |
| Lindi | Lindi Rural | 326 (155) | 3 | Rural coastal | Moslems | 190,280 | 2,015 |
| Mtwara | Mtwara Rural | 215 (141) | 3 | Rural inland | Moslems | 232,586 | 2,412 |
| Pwani | Rufiji | 178 (135) | 3 | Rural coastal | Moslems | 221,641 | 5,564 |
| Pwani | Mkuranga | 204 (128) | 3 | Rural coastal | Moslems | 231,927 | 3,923 |
| Mtwara | Newala | 214 (114) | 3 | Rural inland | Moslems | 209,622 | 4,367 |
| Lindi | Liwale | 206 (103) | 3 | Rural inland | Moslems | 95,072 | 1,925 |
| Mtwara | Masasi | 168 (56) | 3 | Rural inland | Moslems | 252,978 | 5,299 |
| Lindi | Kilwa | 167 (53) | 3 | Rural coastal | Moslems | 194,578 | 3,099 |
| Lindi | Ruangwa | 241 (51) | 3 | Rural inland | Moslems | 133,715 | 2,824 |
| Morogoro | Morogoro | 178 (46) | 2 | Rural inland | Christians | 292,002 | 20,530 |
| Mtwara | Nanyumbu | 171 (44) | 3 | Rural inland | Moslems | 153,889 | 3,330 |
| Pwani | Kisarawe | 175 (42) | 3 | Rural inland | Moslems | 103,640 | 6,541 |
| Morogoro | Kilombero | 139 (39) | 2 | Rural inland | Christians | 424,358 | 17,495 |
| Lindi | Nachingwea | 253 (32) | 2 | Rural inland | Moslems | 182,051 | 3,886 |
| Dar Es Salaam | Temeke | 49 (21) | 2 | Urban coastal | Moslems | 1,538,075 | 9,185 |
| Lindi | Lindi Urban | 46 (18) | 3 | Urban inland | Moslems | 90,265 | 1,160 |
| Pwani | Kibaha | 123 (17) | 3 | Rural inland | Christians | 74,485 | 5,206 |
| Pwani | Kibaha Urban | 156 (14) | 3 | Urban inland | Christians | 141,658 | 5,932 |
| Mtwara | Mtwara Urban | 25 (11) | 3 | Urban coastal | Moslems | 112,674 | 1,173 |
| Dar Es Salaam | Ilala | 24 (10) | 1 | Urban coastal | Moslems | 1,397,478 | 9,063 |
| Dar Es Salaam | Kinondoni | 52 (8) | 2 | Urban coastal | Moslems | 1,956,992 | 9,506 |
| Morogoro | Morogoro Urban | 38 (7) | 2 | Urban inland | Christians | 335,102 | 8,297 |
| Mtwara | Masasi Urban | 35 (4) | 3 | Urban inland | Christians | 106,845 | 2,226 |
| Morogoro | Ulanga | 128 (2) | 2 | Rural inland | Christians | 281,354 | 13,980 |

**Reference**:

1. National Bureau of Statistics. The 2012 Population and Housing Census. Dar Es Salaam; 2012. Available: http://www.nbs.go.tz/index.php/en/census-surveys/population-and-housing-census

2. Sambo M, Mtema Z, Cleaveland S, Sikana L, Lushasi K, Hampson K, et al. Estimating the Size of Dog Populations in Tanzania to Inform Rabies Control. Vet Sci. 2018;5: 77. doi:10.3390/vetsci5030077
